# Subthalamic deep brain stimulation under general anesthesia and neurophysiological guidance while on dopaminergic medication: comparative cohort study

**DOI:** 10.1007/s00701-018-3473-4

**Published:** 2018-02-02

**Authors:** Mohammed Jamil Asha, Benjamin Fisher, Jamilla Kausar, Hayley Garratt, Hari Krovvidi, Colin Shirley, Anwen White, Ramesh Chelvarajah, Ismail Ughratdar, James A. Hodson, Hardev Pall, Rosalind D. Mitchell

**Affiliations:** 1 Department of Neurosurgery, Queen Elizabeth Hospital, University of Birmingham, Mindelson Way, Birmingham, B15 2TH UK; 2 Department of Neuroanaesthesia, Queen Elizabeth Hospital, University of Birmingham, Mindelson Way, Birmingham, B15 2TH UK; 3 Department of Neurophysiology, Queen Elizabeth Hospital, University of Birmingham, Mindelson Way, Birmingham, B15 2TH UK; 4 Department of Statistics, University of Birmingham, Mindelson Way, Birmingham, B15 2TH UK; 5 School of Medicine, University of Birmingham, Mindelson Way, Birmingham, B15 2TH UK

**Keywords:** Subthalamic nucleus deep brain stimulation, Parkinson disease, Dopaminergic therapy, Microelectrode recording, General anesthesia

## Abstract

**Objectives:**

The authors have previously reported on the technical feasibility of subthalamic nucleus deep brain stimulation (STN DBS) under general anesthesia (GA) with microelectrode recording (MER) guidance in Parkinsonian patients who continued dopaminergic therapy until surgery. This paper presents the results of a prospective cohort analysis to verify the outcome of the initial study, and report on wider aspects of clinical outcome and postoperative recovery.

**Methods:**

All patients in the study group continued dopaminergic therapy until GA was administered. Baseline characteristics, intraoperative neurophysiological markers, and perioperative complications were recorded. Long-term outcome was assessed using selective aspects of the unified Parkinson’s disease rating scale motor score. Immediate postoperative recovery from GA was assessed using the “time needed for extubation” and “total time of recovery.” Data for the “study group” was collected prospectively. Examined variables were compared between the “study group” and “historical control group” who stopped dopaminergic therapy preoperatively.

**Results:**

The study group, *n* = 30 (May 2014–Jan 2016), were slightly younger than the “control group,” 60 (51–64) vs. 64 (56–69) years respectively, *p* = 0.043. Both groups were comparable for the recorded intraoperative neurophysiological parameters; “number of MER tracks”: 60% of the “study group” had single track vs. 58% in the “control” group, *p* = 1.0. Length of STN MER detected was 9 vs. 7 mm (median) respectively, *p* = 0.037. A trend towards better recovery from GA in the study group was noted, with shorter “total recovery time”: 60 (50–84) vs. 89 (62–120) min, *p* = 0.09. Long-term improvement in motor scores and reduction in l-dopa daily equivalent dose were equally comparable between both groups. No cases of dopamine withdrawal or problems with immediate postop dyskinesia were recorded in the “on medications group.” The observed rate of dopamine-withdrawal side effects in the “off-medications” group was 15%.

**Conclusions:**

The continuation of dopaminergic treatment for patients with PD does not affect the feasibility/outcome of the STN DBS surgery. This strategy appears to reduce the risk of dopamine-withdrawal adverse effects and may improve the recovery in the immediate postoperative period, which would help enhance patients’ perioperative experience.

## Introduction

The role of subthalamic deep brain stimulation is well established for medically intractable idiopathic Parkinson’s disease. In addition to its obvious benefits in improving selective aspects of motor function, namely rigidity and bradykinesia [9, 15, 27], subthalamic nucleus deep brain stimulation (STN DBS) is proposed to have wide ranging advantages including a significant reduction in PD medications, significant improvement in quality of life, and non-motor functions and more significantly a distinct survival benefit [11, 16, 20, 25].

The technique of STN DBS has evolved over time reflecting the technical advancement in the field. The deployment of microelectrode recording for target localization can be credited for facilitating the adoption of general anesthesia effectively eliminating the need for “awake” macro-stimulation in many functional units. The authors have previously reported their experience with performing these procedures under general anesthesia [13, 24]. This approach is proposed to be a better alternative to “awake surgery” with comparable clinical outcome and safety profile [2, 14, 18].

This technique has raised further questions about the need to stop dopaminergic therapy preoperatively in patients who are undergoing DBS insertion under general anesthesia (GA) and microelectrode recording (MER) guidance. In a preliminary observational study, the authors have recently reported comparable clinical outcome and no added technical difficulty in a small cohort of patients who inadvertently continued their treatment up until the time of surgery [3]. The retrospective study focused mainly on the feasibility of MER recording with dopaminergic medications. Clinical outcome measures were secondarily examined as surrogates for the accuracy of electrode placement.

These positive findings led the authors to change the perioperative medication policy and allowed all PD patients to continue on their regular dopaminergic medications until surgery. Parameters of clinical improvement, postoperative recovery time, perioperative complications, and technical aspects of the procedures were recorded prospectively to verify the previously reported outcomes and examine the effect of dopaminergic therapy on wider aspects of the perioperative care.

## Patients and methods

The study examined a cohort of idiopathic PD patients treated with bilateral STN DBS under GA with MER guidance over the period April 2014–January 2016. All patients were allowed to continue their medications up until surgery, the “new protocol,” *n* = 30. All collected data for this group was done prospectively.

This was compared to a similar cohort of patients who underwent the same procedure in the last 18 months under the old protocol (retrospective data), i.e., with dopaminergic treatment withheld the night before surgery as a historical control group, the “old protocol” *n* = 26.

Data for the study group was collected prospectively by two DBS specialist nurses. The same variables were examined from retrospective data for the historical control group. The examined variables were chosen to address three key questions related to the effectiveness of the procedure and its technical feasibility:

1. Did the continuation of dopaminergic treatment preclude or practically interfere with the intraoperative MER mapping of the STN?
2. Did it affect patients’ recovery in the immediate postoperative period?
3. Did it affect the long-term clinical outcome?

To adequately assess these three main aspects, the following endpoints were examined: intraoperative neurophysiology markers (number of tracks (NOTs) required to obtain satisfactory STN MER and length of recording (LOR) for the detected STN MER). The recovery time in the post-anesthesia recovery unit was recorded prospectively and this was divided into time needed for extubation and time needed for patients to fully recover and be deemed suitable for discharge from the recovery area to the ward. Long-term clinical outcome was assessed using aspects of the unified Parkinson’s disease rating scale (UPDRS) motor score (Hoehn and Yahr scores, number of awake hours spent in “Off” state, and the proportion of the “On” hours spent with dyskinesia) as well as the reduction in the l-dopa equivalent daily dose (LEDD). These clinical outcomes were prospectively recorded at 6 months postoperatively. The examined parameters were compared between the two study groups.

To examine “recovery” in the immediate postoperative stage, two variables were recorded: “time for extubation” and “time for recovery.” The attending anesthetist recorded the time of termination of GA on the anesthetic charts. Subsequently, the time required for extubation (extubation time) and the time spent in the post-anesthesia recovery suite (recovery time) were recorded by the theater staff. This was done prospectively for the study group and compared to the retrospectively retrieved data for the control group. The same comparison was done for any recorded adverse events during hospital stay. The authors defined dopamine-withdrawal effects to include freezing, anxiety, panic attacks, sweating, nausea, generalized pain, fatigue, dizziness, and increased respiratory complications and urinary disturbances—these might be directly related to increased rigidity and poor mobility while off medication.

### Statistical analysis

Power calculations (assuming 80% power and alpha value of 5%) showed that the number of subjects required to make the detected difference in “extubation time” and “recovery time” statistically significant was 837 patients per group and 77 patients per group respectively. Recruiting such a large number was not practically feasible.

Ordinal and continuous variables were compared using Mann-Whitney tests and reported as medians with interquartile ranges. Dichotomous variables were compared between the groups using Fisher’s exact tests. The LOR and NOTs were recorded separately for the left and right sides of the brain. Hence, the analysis was performed separately for each side, as well as for the total of both sides. The NOTs was dichotomized into 1 vs. > 1 for the left/right side analysis, and 2 vs. > 2 for the analysis of the totals.

The examined clinical parameters were compared pre- and postoperatively for the new protocol group using Wilcoxon’s tests, to identify significant changes over time.

All analyses were performed using IBM SPSS 22 (IBM Corp. Armonk, NY), with *p* < 0.05 deemed to be indicative of statistical significant throughout.

## Results

The clinical indications and preoperative selection process was in line with the authors’ standard practice and remained the same for both groups. The surgical technique for STN DBS under GA with MER guidance was also identical for both groups. This was described by the authors previously [3].

### Baseline characteristics

In total, 56 patients were included (median age of 62 years, 68% male), of whom 26 were on the old protocol and 30 on the new protocol. Patient demographics and baseline characteristics were compared between the two groups, Table 1. Patients on the old protocol were found to be older than those on the new protocol (median: 64 vs. 60 years, *p* = 0.043). No significant differences in gender distribution (*p* = 0.779), duration of PD (*p* = 0.603), or the preoperative LEDD at time of surgery were detected between the two groups.

**Table 1:** Comparison between old and new protocols

	Old (*N* = 26)	New (*N* = 30)	*p* value
Age (year)	64 (56–69)	60 (51–64)	*0*.*043*
Gender (male)	17 (66%)	21 (70%)	0.779
Duration of PD (years)	11 (8–14)	12 (9–15)	0.603
LEDD	744 (525–3591)	1221 (1000–1640)	0.650

Data reported as median (IQR), with *p* values from Mann-Whitney tests, or *N* (%), with *p* values from Fisher’s exact tests, as applicable.

Italicized *p* values are significant at *p* < 0.05

### Intraoperative neurophysiology recording markers: LOR and number of MER tracks

No significant difference was detected between the two groups for the total NOTs used, with 60% in the new protocol group, and 58% of patients in the old protocol requiring only one track on either side of the brain to detect satisfactory STN MER (*p* = 1.000). Analysis of the total LOR found this to be significantly higher in the new protocol group, with a median of 9 vs. 7 mm (*p* = 0.037). Breaking this down by the side found that this significant difference was mainly driven by the left side, with a median LOR of 4 mm in the new protocol, compared to 3 mm in the old protocol (*p* = 0.011), Table 2.

**Table 2:** Comparison of LOR and number of tracks between old and new protocols

	Old (*N* = 26)	New (*N* = 30)	*p* value
Left LOR	3 (3–4)	4 (3–5)	*0*.*011*
Right LOR	4 (3–5)	4 (3–6)	0.186
Number of tracks left (> 1)	6 (23%)	11 (37%)	0.384
Number of tracks right (> 1)	8 (31%)	4 (13%)	0.191
Total number of tracks (> 2)	11 (42%)	12 (40%)	1.000

Data reported as median (IQR), with *p* values from Mann-Whitney tests, or *N* (%), with *p* values from Fisher’s exact tests, as applicable

Italicized *p* values are significant at *p* < 0.05

### Postoperative recovery parameters: time for recovery and dopamine-withdrawal side effects

A comparison between the two groups suggested better “recovery time” in the new protocol compared to the old protocol cohort although this fell short of statistical significance. However, a significant difference in the recorded perioperative complications was evident with four patients in the old cohort reported to have mainly respiratory and urinary complications compared to none in the new cohort, Table 3.

**Table 3:** Postoperative recovery times

	Overall (*N* = 56)	Old (*N* = 26)	New (*N* = 30)	*p* value
Extubation time (mins)	23 (15–37)	25 (15–44)	22 (15–30)	0.621
Recovery time (mins)	73 (53–113)	89 (62–120)	60 (50–84)	0.096

Data reported as median (IQR), with *p* values from Mann-Whitney tests

The continuation of dopaminergic medications might be associated with reduction in the length of hospital stay. The average hospital stay for old cohort was 3.5 days compared to 2.5 days in the new cohort, *p* > 0.05.

### Long-term clinical outcome: motor scores and reduction of LEDD

Practical aspects of the UPDRS motor scores were selected for clinical outcome assessment and were recorded preoperatively at the time of enlisting patients for the surgical procedure and subsequently at 6 months postoperatively. The authors used the formula previously reported in the same unit [26] for calculating the LEDD. The reduction in LEDD is widely accepted as a surrogate marker for clinical improvement after DBS and appears to show good outcome in the new protocol cohort. The comparison of the preoperative and postoperative scores for the examined endpoints suggests a clear clinical improvement in the study group, Table 4. This is in line with previously reported data for patients who underwent the procedure after withholding their medications [13].

**Table 4:** Pre- and postoperative comparisons in the new protocol group

	Preoperative	Postoperative	*p* value
Hoehn-Yahr	2 (2–3)	1 (1–2)	< *0*.*001*
“Off” awake hours	5 (2–7)	0 (0–1)	< *0*.*001*
Dyskinesia % of “On” hours	30 (10–50)	0 (0–0)	< *0*.*001*
LEDD	1221 (1000–1640)	553 (360–728)	< *0*.*001*

Data reported as median (IQR), with *p* values from Wilcoxon’s tests

Italicized *p* values are significant at *p* < 0.05

## Discussion

The value of MER remains a subject of debate for many authors [12] with some proposing that direct anatomical targeting is good enough [29]; however, MER advocates have reported significant adjustments of “anatomical” targeting using MER without significant added complications [6, 22, 23].

The cessation of dopaminergic medications preoperatively is a practical necessity for awake macro-stimulation but in the context of MER-guided DBS insertion under GA, it lacks a solid evidence foundation.

However, some neurophysiological studies have previously suggested that dopaminergic therapy might interfere with beta-oscillations potentially masking the pathological STN activity [21]. This could make obtaining a satisfactory STN recording intraoperatively more difficult and add the associated risks of extra brain penetrations. This consideration appears to be more of a theoretical risk as it is suggested that the electrical activity picked up by MER is not solely comprised of B-range oscillations but includes other local field potentials of variable frequencies [7, 8, 28]. Indeed these local field potentials remain even after DBS insertion for a considerably long time [1].

The authors have previously suggested the feasibility of obtaining good-quality microelectrode recording of the STN in patients who continue on dopaminergic treatment until surgery [3, 4]. The choice of LOR and NOTs serves as practical surrogates for the quality of recording and correlates with the debated risk of increased intra-parenchymal hemorrhage with added brain penetrations for the MER electrodes [5]. In this study, the authors report a statistically significant difference in LOR between the two study groups in favor of the study group. The significance of this result is uncertain as this might reflect an artifact due to slightly higher degree of brain atrophy (age-related) in the “older” cohort of the control group leading to the risk of brain shift after opening the dura and loosing CSF [10].

The continuation of dopaminergic therapy combined with the “lesioning effect” from the DBS electrode placement might be argued to increase the risk of postoperative dyskinesia [17, 19]. The authors’ experience from both studies have not shown evidence of this problem.

The reported effect of dopaminergic medication continuation on the “recovery time” in this study fell short of statistical significance. This, however, should be taken in context of the study limitations, mainly the small cohort size and the fact that the data collection for the study group was done prospectively but compared to a retrospective data for the historical control group. This can be argued to compromise the internal validity of such comparison.

In considering these limitations, the authors acknowledge that the study was statistically underpowered to detect significant difference in the recovery times between the two groups based on the required large sample size as calculated previously. However, despite the lack of statistical significance, the authors suggest that the effect of continuing dopaminergic therapy may be reflected beyond the immediate post-GA recovery with earlier mobilization and lower risk of respiratory and urinary complications. These would be related to increased rigidity and poor mobility in the perioperative period.

The authors also acknowledge the fact that the study design might be challenged by the use of historical controls. This might be suggested to introduce some inconsistency in the way that the time for intubation or time for recovery was recorded. Such inconsistencies, if exist, would be inconsolable. Nevertheless, the recording of these times is an established practice in the authors’ institute as the time for cessation of GA is automatically logged on the system when the anesthetic agent is switched off. The same practice follows when the patient is extubated.

The arrival time to the post-GA Recovery suite as well as the time when the patient was deemed ready to leave are routinely recorded on an electronic log.

In view of these facts, the authors believe—despite the obvious limitation of retrospective data in the historical group—that such comparison remains valid as the data was collected in a controlled fashion as standard practice in all cases prospective or historical. Moreover, the data collection for these variables is not done by the surgical team but rather by independent recovery/theater staff who are not concerned with the specific details of the surgical procedure or the status of preoperative medications.

The improvement in postoperative recovery is further suggested by the apparent reduction in the length of hospital stay (average hospital stay for old cohort 3.5 days vs. 2.5 days in the new cohort, *p* > 0.05).

In the authors’ current practice, patients are admitted on the morning of surgery rather than the night before which was previously required due to the anticipated reduction in mobility from withdrawal of medication. It is suggested that the avoidance of dopamine withdrawal and its consequential risks will allow patients to be discharged home sooner.

The long-term clinical outcome has been examined using standard measures utilized by the UPDRS scale. These clearly show good outcome for all patients effectively reflecting good placement of the DBS electrodes in line with the reported literature.

The authors are encouraged by these positive findings which seem to support the desirable effect of keeping PD patients on their dopaminergic treatment up until the time of surgery.

In considering the limitations of this study, the authors highlight the limited cohort size and the lack of controlled design in this observational comparative study. The fact that the new protocol cohort had an average younger age group might have resulted in an artifact with slightly better LOR for microelectrode recordings in the study group.

Despite these limitations, the authors believe that the results of this prospective study confirm that it is possible to achieve good electrophysiological recording without withholding dopaminergic medication. There is further support of the initial observation that dopaminergic therapy continuation leads to a better perioperative experience and appears to project into sustained good clinical outcome with lower complications rates.

## Conclusions

Subthalamic deep brain stimulation under GA can be effectively performed with intraoperative MER guidance in patients on dopaminergic therapy. The quality of STN microelectrode recordings and the clinical effectiveness of the procedure appear not to be compromised by continuing the medications.

Keeping patients on their regular therapy appears to carry lower risk of postoperative complications (due to drug-withdrawal effects). It provides good long-term clinical outcome and potentially smoother recovery from general anesthesia.

Based on the authors’ current experience, the cessation of dopaminergic therapy for this group of patient is not justifiable and might compromise the clinical outcome.

## Abbreviations

CSF: Cerebrospinal fluid
DBS: Deep brain stimulation
GA: General anesthesia
IQR: Interquartile range
LEDD: l-dopa equivalent daily dose
LOR: Length of recording
MER: Microelectrode recording
NOTs: Number of tracks
PD: Parkinson’s disease
STN: Subthalamic nucleus
